# ASO Author Reflections: Toward Improved Selection of Patients for Cytoreduction and HIPEC: Identification of Prognostic Factors for Patients with Colorectal Peritoneal Metastases

**DOI:** 10.1245/s10434-018-6842-6

**Published:** 2018-10-09

**Authors:** Nina R. Sluiter, Jurriaan B. Tuynman

**Affiliations:** Department of Surgery, Amsterdam UMC, Vrije Universiteit Amsterdam, Amsterdam, The Netherlands

## Past

Cytoreduction and hyperthermic intraperitoneal chemotherapy (HIPEC) improve survival of patients with colorectal peritoneal metastases (PM).^1^ Careful patient selection is pivotal because this treatment is associated with substantial morbidity, but current selection criteria fail to discriminate sufficiently between patients who will and those who will not benefit. Next to preoperative imaging, which has limited accuracy for detection of PM, selection is based largely on intraoperative assessment of the peritoneal cancer index (PCI).^2^ However, selection should ideally take place before surgery.

Therefore, identification of practical clinicopathologic prognostic variables is key to optimize treatment for patients with PM. Patients who experience PM despite previous adjuvant chemotherapy after primary tumor resection are thought to benefit less from cytoreduction and HIPEC, and several studies and guidelines exclude these patients from a potentially curative HIPEC treatment.^3^^,^^4^ However, no data are available to support clinical decision making for patients with PM. This study evaluated potential clinicopathologic prognostic variables for 175 patients, with special interest in early peritoneal recurrence after previous chemotherapy.

## Present

This study evaluated all relevant clinicopathologic variables potentially associated with oncologic outcome in a large prospectively obtained cohort of 175 patients who had colorectal PM and were treated with cytoreduction and HIPEC.^5^ Interestingly, the most significant risk factor for worse disease-free and overall survival was early peritoneal recurrence after primary tumor treatment. In particular, patients who had recurrence within 1 year after adjuvant chemotherapy experienced worse survival than patients who did not receive adjuvant chemotherapy or patients with peritoneal recurrence after 1 year. The only other significant factor associated with worse disease-free and overall survival in the multivariate analysis was a high PCI. No other variables, including primary tumor stage, were associated with survival, indicating that the current results were not biased by treatment allocation. The current findings are of clinical significance and support consideration of both PCI and early peritoneal recurrence after adjuvant therapy for patient selection before cytoreduction and HIPEC, thereby minimizing unnecessary exposure to an invasive procedure with high morbidity rates.

## Future

Future guidelines should incorporate the identified clinicopathologic prognosticators into clinical practice. Before clinical implementation, however, these variables require further prospective validation. A combined effort, with prospectively collected consecutive data from patients with PM, is key for this purpose. Currently, ongoing clinical trials with patients who have colorectal PM are addressing perioperative systematic chemotherapy, the additional value of HIPEC versus cytoreduction alone, and novel chemotherapeutic HIPEC agents. Future research to optimize treatment of patients with PM should focus on identification of molecular tumor characteristics that could improve patient selection. In addition, the substantial progress regarding the use of blood samples as liquid biopsies could provide prognostic (epi-)genetic information. Knowledge of tumor biology not only could help in selecting the right patients, but also could provide guidance regarding the optimal chemotherapeutic drug for the HIPEC treatment. Ultimately, a personalized profile consisting of both clinical and molecular characteristics will provide a way toward tailored treatment for patients with colorectal PM.
